# Enhanced resistive switching memory characteristics and mechanism using a Ti nanolayer at the W/TaO_*x*_ interface

**DOI:** 10.1186/1556-276X-9-125

**Published:** 2014-03-17

**Authors:** Amit Prakash, Siddheswar Maikap, Hsien-Chin Chiu, Ta-Chang Tien, Chao-Sung Lai

**Affiliations:** 1 Department of Electronic Engineering, Chang Gung University, Tao-Yuan 333, Taiwan; 2 Material and Chemical Research Laboratories, Industrial Technology Research Institute, Hsinchu 310, Taiwan

**Keywords:** Resistive switching, W/TaO_*x*_, Ti nanolayer, Oxygen ion migration, Nanofilament

## Abstract

Enhanced resistive memory characteristics with 10,000 consecutive direct current switching cycles, long read pulse endurance of >10^5^ cycles, and good data retention of >10^4^ s with a good resistance ratio of >10^2^ at 85°C are obtained using a Ti nanolayer to form a W/TiO_*x*_/TaO_*x*_/W structure under a low current operation of 80 μA, while few switching cycles are observed for W/TaO_*x*_/W structure under a higher current compliance >300 μA. The low resistance state decreases with increasing current compliances from 10 to 100 μA, and the device could be operated at a low RESET current of 23 μA. A small device size of 150 × 150 nm^2^ is observed by transmission electron microscopy. The presence of oxygen-deficient TaO_*x*_ nanofilament in a W/TiO_*x*_/TaO_*x*_/W structure after switching is investigated by Auger electron spectroscopy. Oxygen ion (negative charge) migration is found to lead to filament formation/rupture, and it is controlled by Ti nanolayer at the W/TaO_*x*_ interface. Conducting nanofilament diameter is estimated to be 3 nm by a new method, indicating a high memory density of approximately equal to 100 Tbit/in.^2^.

## Background

Resistive switching random access memories (RRAM) with simple metal-insulator-metal stacks are under intensive investigation owing to their great promise for use in next-generation memory applications [1-5]. However, their nonuniformity in switching, low yield, and unclear switching mechanism hinder their practical realization. RRAM devices with simple composition, easy fabrication process, and good 3D integration compatibility will be needed in the future. Methods such as doping, formation polarity control, bottom electrode modification, nanocrystal insertion, and interfacial engineering have recently been investigated to improve the characteristics of resistive switching memory [6-10]. Among other important switching materials such as TiO_*x*_[11,12], NiO_*x*_[13-15], HfO_*x*_[10,16-18], ZrO_*x*_[19-27], Na_0.5_Bi_0.5_TiO_3_[28]_,_ SrTiO_3_[29], ZnO [30,31], GeO_*x*_[32], and SiO_*x*_[33], tantalum oxide (TaO_*x*_) is one of the most promising choices for future RRAM applications. However, TaO_*x*_-based RRAM devices are infrequently reported [5,34-39]. Terai et al. [37] used a TiO_2_ layer in a Ru/Ta_2_O_5_/TiO_2_/Ru stack with good thermal stability. Ninomiya et al. [38] reported an Ir/Ta_2_O_5−*δ*_/TaO_*x*_/TaN structure, and Lee et al. [5] reported a Pt/Ta_2_O_5−*x*_/TaO_2−*x*_/Pt crossbar structure with two layers of TaO_*x*_ and at least one of the inert electrodes such as Ru, Ir, and Pt. Generally, many researchers use one inert electrode to improve the performance of resistive switching memory [5,39]; however, tungsten (W) as both bottom and top electrodes in a W/TiO_*x*_/TaO_*x*_/W structure has not yet been reported. Furthermore, the RRAM devices with low current operation (<100 μA) is also a challenging issue. In this work, a resistive switching memory device using a Ti nanolayer at the W/TaO_*x*_ interface and enhanced memory characteristics such as excellent 10,000 consecutive stable dc switching cycles, long read pulse endurance of >10^5^ cycles, and good data retention of 10^4^ s at 85°C with a large resistance ratio of >10^2^ under a low compliance current (CC) of 80 μA are reported. Furthermore, the device can be operated with a small ‘RESET’ current of 23 μA. For comparison, the W/TaO_*x*_/W memory device is also fabricated. The device size of 150 × 150 nm^2^ is observed using a high-resolution transmission electron microscope (HRTEM). The thicknesses of TiO_*x*_ and TaO_*x*_ nanolayers are 3 and 7 nm, respectively. The presence of oxygen-deficient TaO_*x*_ conducting filaments is investigated by Auger electron spectroscopy (AES) before and after switching of the memory devices. The switching mechanism of the oxygen ion migration owing to a lower barrier height of electrons is investigated, and a filament diameter of approximately equal to 3 nm is calculated using a new method also reported in this work. Considering a small filament diameter, a high memory density of approximately equal to 100 Tbit/in.^2^ could be designed in the future.

## Methods

W/Ti/TaO_*x*_/W-structured (device S1) and W/TaO_*x*_/W-structured (device S2) resistive switching memory stacks were fabricated. A small via size of 150 × 150 nm^2^ was etched into the SiO_2_ on W bottom electrode (BE), which was about 100 nm in thickness. Standard photolithography and dry etching processes were used to open the via holes for the RRAM devices. The photoresist (PR) was coated and opened on active and top electrode (TE) regions for lift-off process. Then, a high-*κ* Ta_2_O_5_ film with a thickness ($t_{{\mathrm{Ta}}_{2}O_{5}}$) of approximately equal to 7 nm was deposited by an e-beam evaporator, followed by the sequential deposition of a thin (approximately equal to 3 nm) interfacial layer of titanium (Ti) and approximately equal to 200-nm-thick W layer as a TE by radio-frequency (rf) sputtering. The W and Ti targets were used. Initial vacuum was approximately 10^−5^ Torr. Argon gas (Ar) with a flow rate of 25 sccm and deposition power of 100 W was used to deposit W. The W deposition rate was 10 nm/min. For Ti deposition, Ar with a flow rate of 15 sccm and deposition power of 150 W were used. The Ti deposition rate was approximately 6.5 nm/min. For device S2, no Ti layer was deposited. The final devices were obtained after a lift-off process. Memory device structure and thicknesses of all layers were observed by transmission electron microscopy (TEM) with an energy of 200 keV. The TaO_*x*_ material was also confirmed by quadrupole secondary ion mass spectroscopy (SIMS; ATOMIKA SIMS 4500, MA-Tek, Hsinchu, Taiwan) which had a high-depth resolution. Primary beam was O^2+^ with an energy of 0.5 keV and analysis area of 37.5 × 37.5 μm^2^. A bias was applied to the TE, and the BE was electrically grounded. Pristine S1 and S2 devices were electroformed by applying positive voltage to the TE before consecutive resistive switching cycle measurements.

## Results and discussion

**Figure 1:**
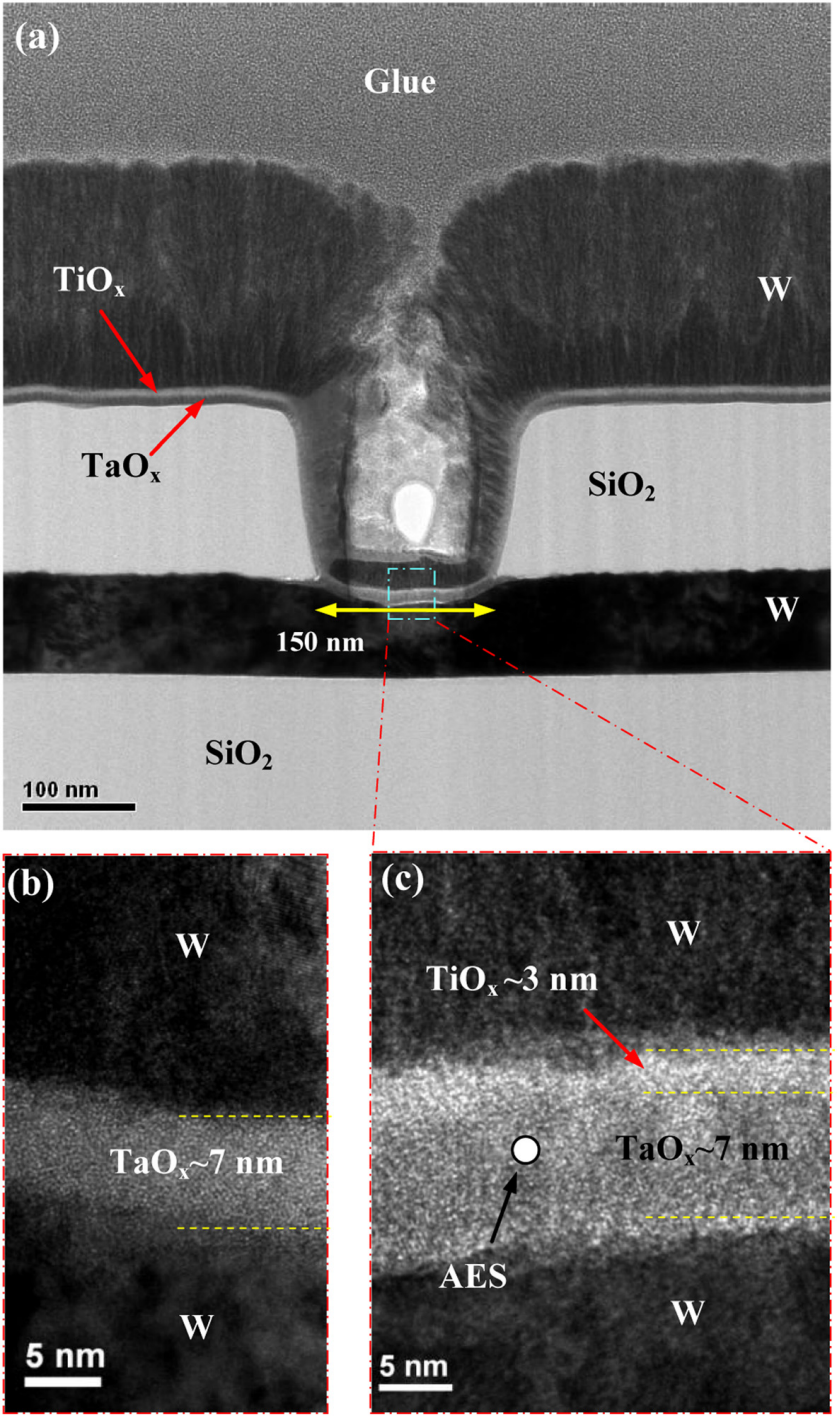
**TEM and HRTEM images of W/TiO**_***x***_**/TaO**_***x***_**/W (S1) and W/TaO**_***x***_**/W (S2) structures. (a)** TEM image of fabricated W/TiO_*x*_/TaO_*x*_/W (S1) structure. HRTEM images of **(b)** W/TaO_*x*_/W (S2) and **(c)** W/TiO_*x*_/TaO_*x*_/W (S1) structures.

**Figure 2:**
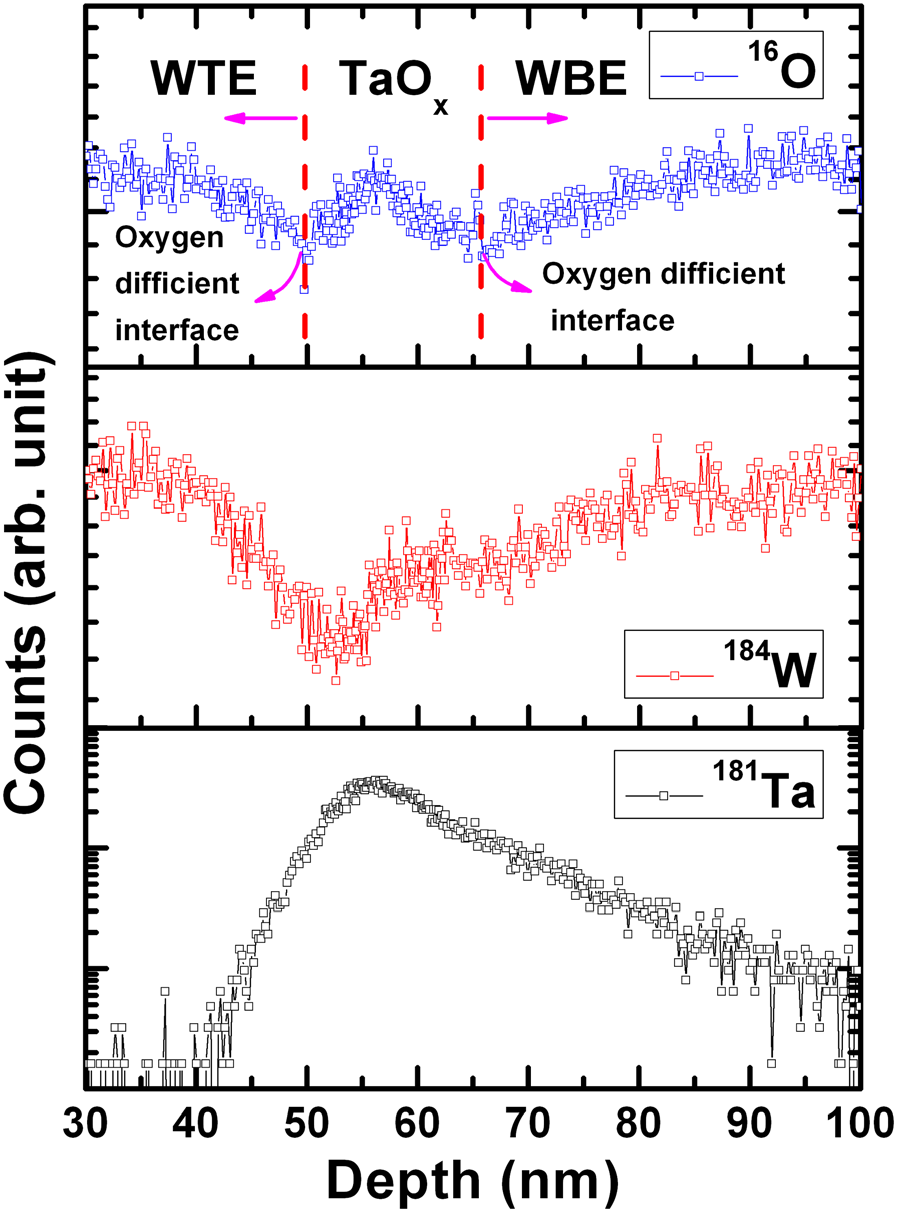
**SIMS depth profile of W/TaO**_***x***_ **/W (S2) structure.**

**Figure 3:**
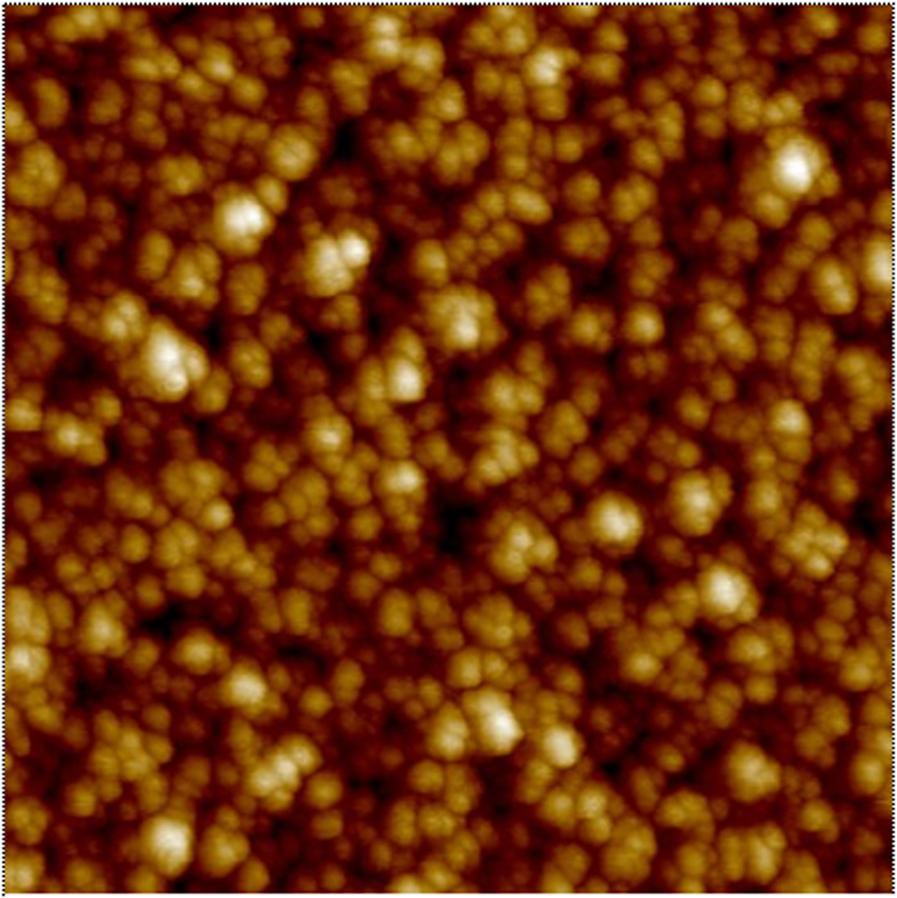
**AFM image for surface roughness.** The surface roughness of 200-nm-thick W layer on SiO_2_/Si substrate is approximately 2.8 nm.

Figure 1a shows a typical cross-sectional TEM image of the W/TiO_*x*_/TaO_*x*_/W structure. The device size is 150 × 150 nm^2^. HRTEM images of the S2 and S1 devices are shown in Figure 1b,c. The thicknesses of the TiO_*x*_ and TaO_*x*_ layers are approximately 3 and 7 nm, respectively, and both films show an amorphous characteristic. The film deposited by rf sputtering is not a conformal deposition. Therefore, the TiO_*x*_ layer can be seen clearly on outside and active regions of the via hole (Figure 1a,c); however, this layer is not observed clearly on the sidewall of the via hole. It is also obvious that the switching material on the sidewall is not necessary for switching properties of the RRAM devices because the electrons will find least path to move from TE to BE. This TiO_*x*_ layer is also confirmed on outside and active region of the device by energy dispersive X-ray spectroscopy (not shown here). Figure 2 shows typical SIMS depth profiles of ^16^O, ^184^ W, and ^181^Ta materials for the S2 sample. The thickness of the TaO_*x*_ layer is about 15 nm; however, this is higher than the deposited film thickness of 7 nm. This is due to the trail effect and surface roughness of W BE, as we can see from the depth of 57 to 65 nm (or approximately 7 nm) of the ^184^ W depth profile. The average surface roughness of 200-nm-thick W layer on SiO_2_/Si substrate is approximately 2.8 nm, which is observed by atomic force microscopy (AFM) with a scan area of 1 × 1 μm^2^, as shown in Figure 3. Therefore, the remaining thickness of approximately 4.2 nm (=7 to 2.8 nm) is coming from the trail effect of the SIMS depth profile. The depth from 50 to 57 nm is the thickness of the TaO_*x*_ layer, which is approximately 7 nm, as shown in Figure 2a,c. It is interesting to note that the TaO_*x*_/W interface is found to be an oxygen-deficient layer, which makes it a more conducting interface. On the other hand, the conducting filament will be formed after breaking the Ta-O bonds in the bulk Ta_2_O_5_ layer rather than the W/TaO_*x*_ interface. This is because the Ta_2_O_5_ layer is more insulating than the W/TaO_*x*_ interface, so the electric field will drop across the Ta_2_O_5_ film rather than the W/TaO_*x*_ interface which probably results multi-filaments or an uncontrolled nanofilament diameter. As Ti removes oxygen from the Ta_2_O_5_ film in the W/TiO_*x*_/TaO_*x*_/W structure, the TaO_*x*_ film becomes more oxygen-deficient, which is vital to achieve an improved resistive switching. Considering Gibbs free energies of TiO_2_, Ta_2_O_5_, and WO_3_ films, which are −887.6, −760.5, and −506.5 kJ/mol, respectively, at 300 K [40], Ti will consume the highest oxygen content owing to its stronger reactivity than those of the other materials, thereby forming a Ta-rich (or defective TaO_*x*_) film. This also prevents oxidation of the W TE at the TaO_*x*_/W interface of device S1 owing to the migration of oxygen from the underlying films towards the Ti film, which contributes to the improved resistive switching memory performance as will be described.

**Figure 4:**
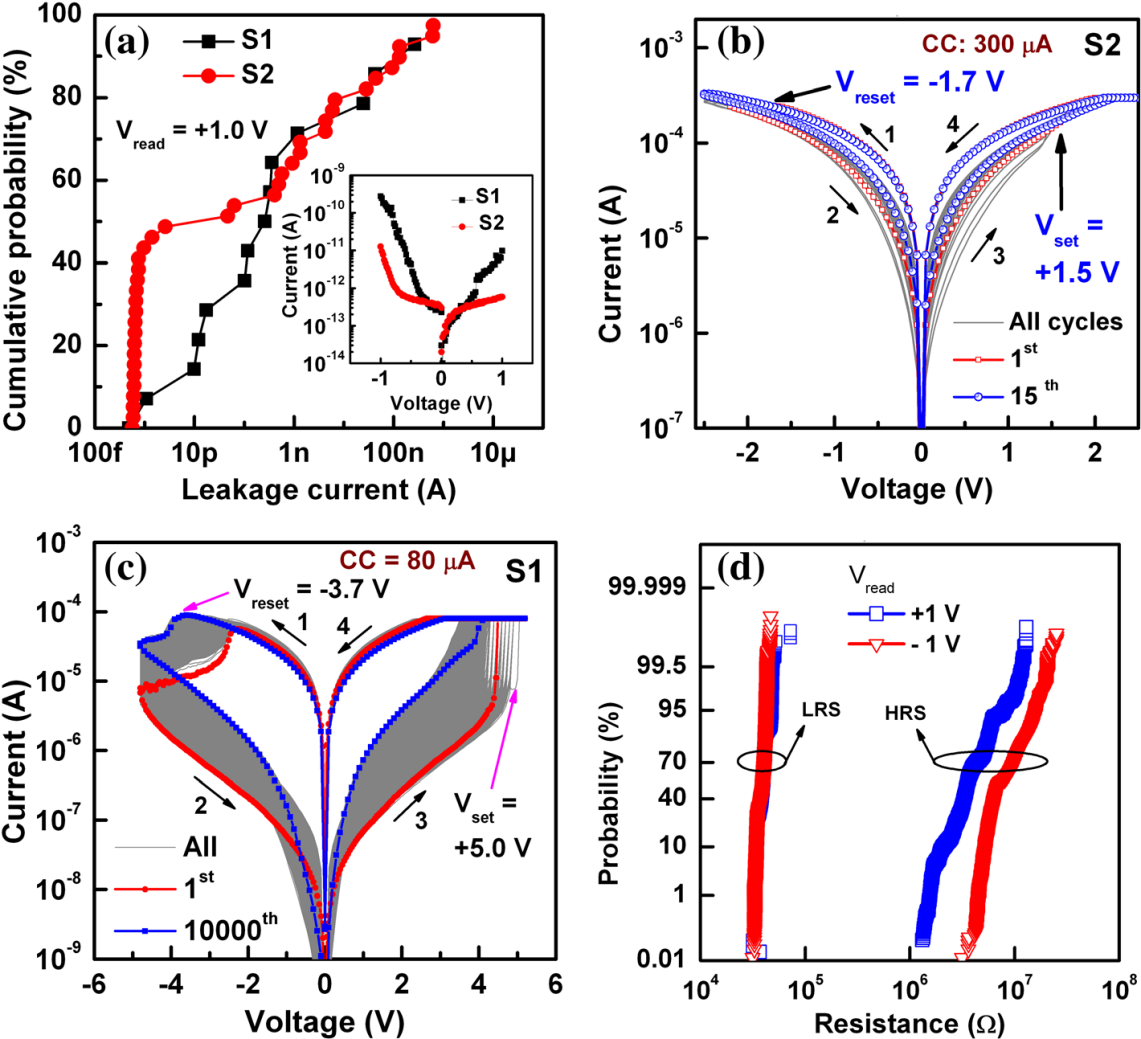
**Cumulative probabilities of leakage currents and LRS/HRS and switching *I*-*V *curves of S1 and S2. (a)** Cumulative probability of leakage currents for S1 and S2 devices with typical via size of 0.8 μm. Inset: leakage current vs. voltage characteristics. Switching *I*-*V* curves of **(b)** S2 and **(c)** S1 devices. The S2 device shows instability after a few cycles, while 10,000 consecutive switching cycles are observed for the S1 device. **(d)** Cumulative probability of LRS and HRS under *V*_read_ of ±1 V for the S1 devices.

**Figure 5:**
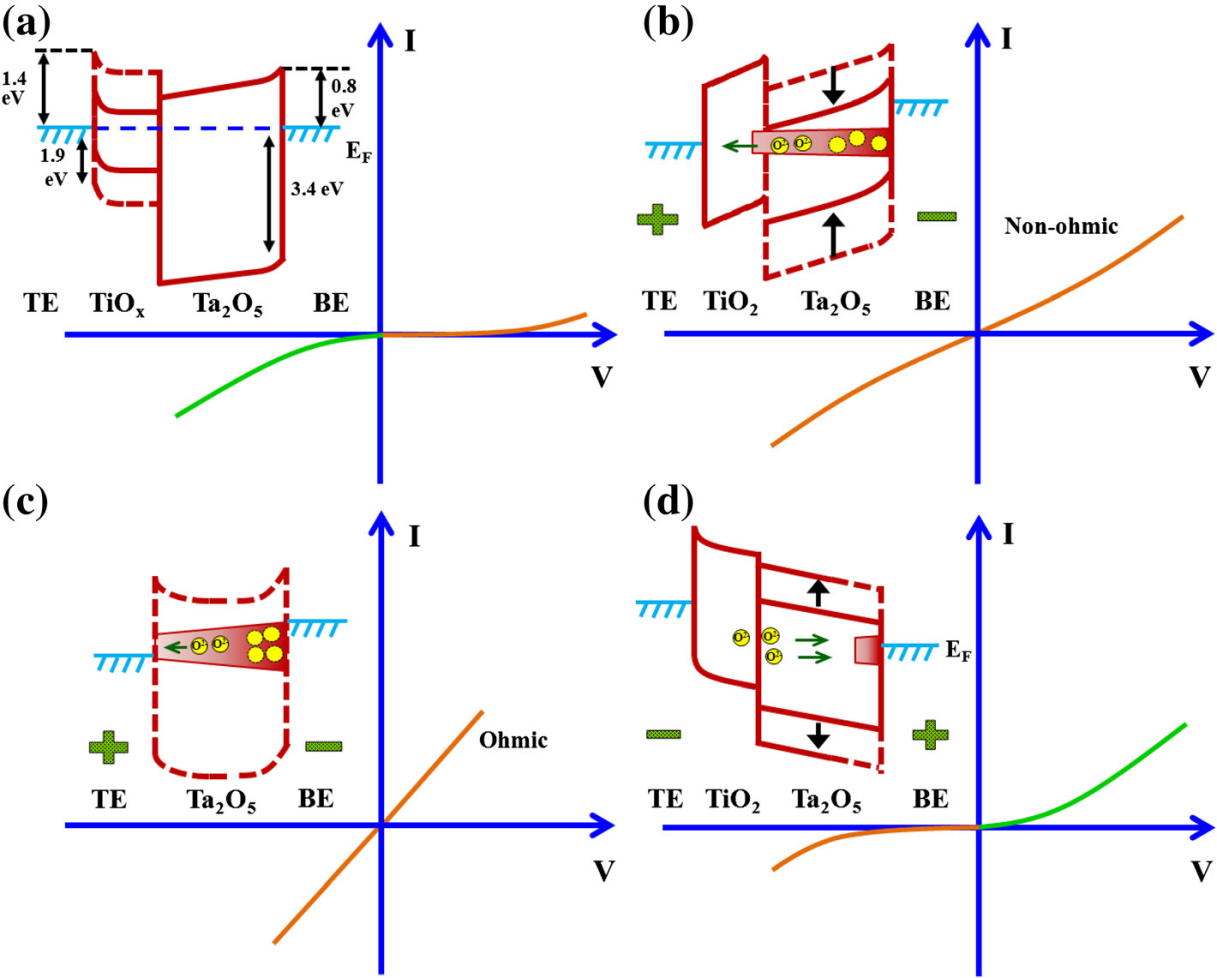
**Schematic illustration of switching mechanism using simple energy band diagrams with *I*-*V *characteristics. (a)** Energy bands of pristine S1 structure device and ideal leakage current. For TiO_2_, the solid line is due to the smaller energy gap of the defective TiO_*x*_ film. *I*-*V* of LRS and corresponding energy bands with conducting filaments for **(b)** S1 and **(c)** S2 devices. For TaO_*x*_, the solid line is due to the lower energy gap caused by the presence of oxygen-deficient filament. The S1 devices show non-ohmic *I*-*V* due to TiO_2_ layer formation at the W/TaO_*x*_ interface. **(d)** Filament oxidation and leakage current at HRS are shown for the W/TiO_*x*_/TaO_*x*_/W devices. Filament formation/rupture is controlled by the TiO_2_ layer due to O^2−^ ion migration.

**Figure 6:**
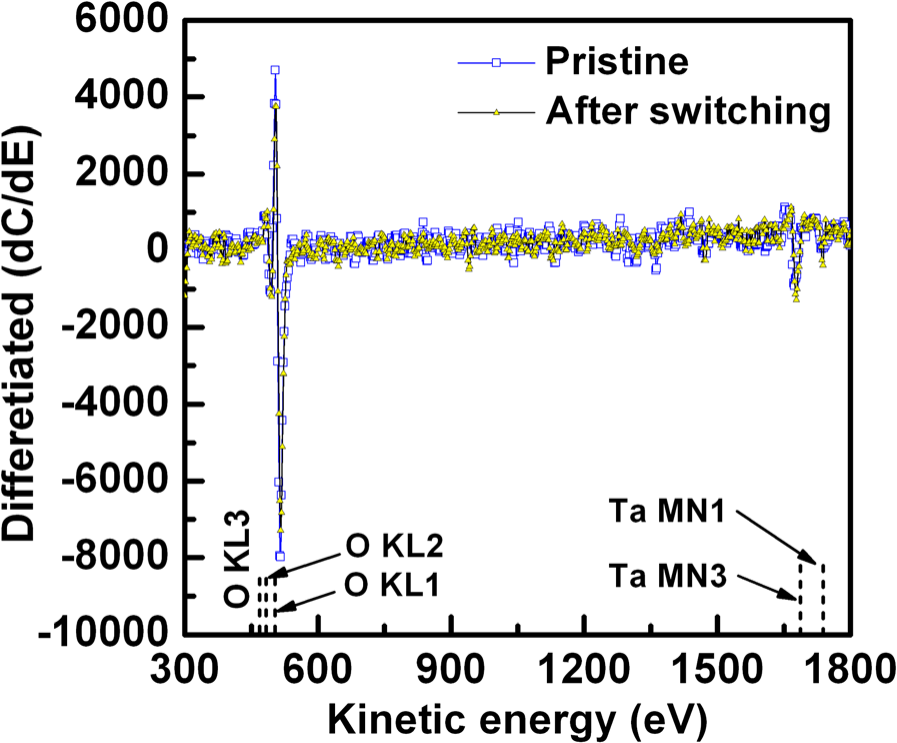
**Differentiated counts with respect to kinetic energy (dC/dE).** AES spectra vs. kinetic energy for pristine and after-switching RRAM devices. The spectra are from a typical via size of 0.4 × 0.4 μm^2^ and measured inside the middle of via regions (open blue square symbols for pristine and yellow solid triangle symbols for switched devices). An oxygen-deficient TaO_*x*_ layer is observed after few switching cycles, confirming oxygen-deficient TaO_*x*_ filament formation after SET.

The leakage current values of most of the S1 pristine devices at a read voltage (*V*_read_) of 1 V are higher than that of the S2 devices because of the presence of more oxygen vacancies in the TaO_*x*_ layer owing to the oxygen-getter nature of the TiO_*x*_ layer (Figure 4a). Typical current-voltage (*I*-*V*) curves (inset of Figure 4a) of both devices were asymmetrical with higher current at a negative voltage (≈281 pA for S1 and ≈ 12.6 pA for S2 at *V*_read_ = −1 V) compared with that measured at a positive voltage (≈9.8 pA for S1 and ≈ 0.6 pA for S2 at *V*_read_ = 1 V). This suggests that the W TE/TaO_*x*_ interface has more oxygen vacancies than the TaO_*x*_/W BE interface, owing to oxygen migration towards W TE during deposition. The ideal leakage current is plotted in Figure 5a and is explained as follows: It is reported that the work function (*Ф*_m_) of W and bandgap (*E*_g_) of amorphous Ta_2_O_5_ and TiO_2_ are 4.55 [41], 4.2 [42], and 3.3 eV [43], respectively. The conduction band offsets of Ta_2_O_5_ and TiO_2_ with Si are 0.3 [44] and 0.9 eV [45], respectively. Taking the electron affinity of Si as 4.05 eV, the electron affinities of Ta_2_O_5_ and TiO_2_ are calculated to be 3.75 and 3.15 eV, respectively. The corresponding energy diagram is shown in Figure 5a as solid lines. Considering that the *E*_g_ of TiO_2_ for the pristine S1 device will be much lower because of oxygen vacancy creation during the deposition of W TE, the band diagram is shown in dotted lines (Figure 5a). In this case, electron injection dominates rather than hole injection because of a lower barrier height for electrons than for holes (0.8 to 1.4 vs. 3.4 eV). Both S1 and S2 devices show bipolar resistive switching behaviors. The S2 device shows few switching cycles with a higher leakage current of ≈ 10 μA at *V*_read_ = 1 V and a higher CC of 300 μA (Figure 4b). In this case, negatively charged oxygen ions (O^2−^) migrate from the switching material towards W TE, and this has a lesser possibility to form an oxygen-rich layer at the W TE/TaO_*x*_ interface, leading to the formation of multi-conduction filaments. In the same way, no resistive switching is observed under negative forming voltage for either the S1 or S2 devices because oxygen ions migrate towards the W BE and permanent breakdown is observed (not shown here). The negative forming will lead to high switching current, which is similar to the W/TaO_*x*_/W structure, and there is no oxygen-rich interfacial layer at the W/TaO_*x*_ interface. This interfacial layer will have series resistance and protect from current overshoot effect. However, the insertion of a thin (≈3 nm) Ti layer in between the W and TaO_*x*_ layers in the S1 device makes a vast difference because Ti can be used as an oxygen reservoir. Moreover, the S1 device exhibits >10,000 consecutive repeatable dc switching cycles with a better resistance ratio of 10^2^ under a low CC of 80 μA (Figure 4c). The transport mechanism follows the trap-charge-controlled space-charge-limited current conduction (not shown here). However, a thicker Ti layer (5 nm) results in unstable switching cycles because it gets more oxygen and behaves as an insulating layer. This may lead to the conducting filament formation/rupture in the TiO_*x*_ layer rather than the TaO_*x*_ layer. It is reported that the TiO_2_ switching layer has a Magneli phase and the memory window is collapsed after a few cycles [46]. That is the reason of having unstable switching using thicker (5 nm) Ti interracial layer. Therefore, thickness optimization is very important and we have chosen those thicknesses of TaO_*x*_ and TiO_*x*_ layers here. The thinnest Ti layer of <3 nm is also not to be used because of direct current flow through this layer. Therefore, the thinner (3 nm) Ti layer will control the current overflow as well as will control the filament diameter. The yield of the S1 device is very high (>95%), while that of the S2 device is very low (approximately 10%). In addition, the S2 device cannot be switched below a CC of 300 μA and shows an ohmic behavior, while the S1 device shows switching even at a low CC of 10 μA (discussed later) with non-ohmic current conduction. The average values and standard deviation/average are found to be 39.7 and 0.11, 38.4 kΩ and 0.08 for low-resistance state (LRS) and 1.9 and 2.11, 8.6 MΩ and 0.43 for high-resistance state (HRS) at *V*_read_ of 1 V and −1 V, respectively (Figure 4d). This suggests that the LRS has a tighter distribution than the HRS because of the formation of the TiO_2_ layer, which will have a higher *E*_g_ than the pristine one. Similarly, the leakage current at *V*_read_ of −1 V is lower than that at +1 V because of the lower electron injection barrier at the TE/TiO_2_ interface than that at the BE/TaO_*x*_ interface after switching. Under ‘SET’, O^2−^ will migrate from TaO_*x*_ towards the TE, resulting in a TiO_2_ layer which controls the conducting vacancy filament diameter in the TaO_*x*_ layer by controlling current overflow and producing a tighter distribution of the LRS. Owing to this series resistance, the S1 devices exhibit non-ohmic-simulated (or nonlinear) ideal current, as shown in Figure 5b, whereas an ohmic current is observed for the S2 devices under SET (Figure 5c). It is true that the conducting filament is formed through the TaO_*x*_ film (Figure 5b,c), which is also confirmed by the AES spectra of the TaO_*x*_ film for pristine and after-switching of the TaO_*x*_-based devices (Figure 6). The differentiated counts with respect to kinetic energy (dC/dE) versus kinetic energy (E) are plotted. The spectrum positions are in the middle of the TaO_*x*_ switching layer with a typical device size of 0.4 × 0.4 μm^2^. Different RRAM devices of pristine and switching were used to get the AES spectra. Even though different devices were used, the spectra of both the pristine (blue open square symbols) and switched (yellow solid triangle symbols) devices were maintained from the same depth. Ta-MN (1,737 and 1,680 eV) and O-KL (468, 483, and 503 eV) are observed, which confirms the formation of a TaO_*x*_ layer. The atomic percentages of Ta-MN3 and O-KL1 are 37.38% and 62.62% for the pristine device and 44.69% and 55.31% for the switched device, respectively. It is believed that the spectra difference is not a variation, and the oxygen ion migration from the TaO_*x*_ switching layer. Due to a small amount of oxygen migration, the difference of the two spectra will be small. The atomic percentages were calculated by using commercial software for AES spectra. Basically, this decrease in oxygen content and increase in Ta content after switching is of the evidence that an oxygen-deficient filament is formed owing to oxygen ion migration as well as the lower energy gap of the TaO_*x*_ layer, as shown by the dotted line in Figure 5b. When negative voltage is applied to the TE, oxygen ions are pushed from the TiO_2_ layer towards the conducting filament where they recombine with oxygen vacancies or oxidize the conducting filament. The device will be in HRS (Figure 5d). Control of oxygen-deficient filament formation and rupture is facilitated by insertion of the thin Ti layer at the TE/TaO_*x*_ interface, which results in repeatable and reproducible resistive switching characteristics.

**Figure 7:**
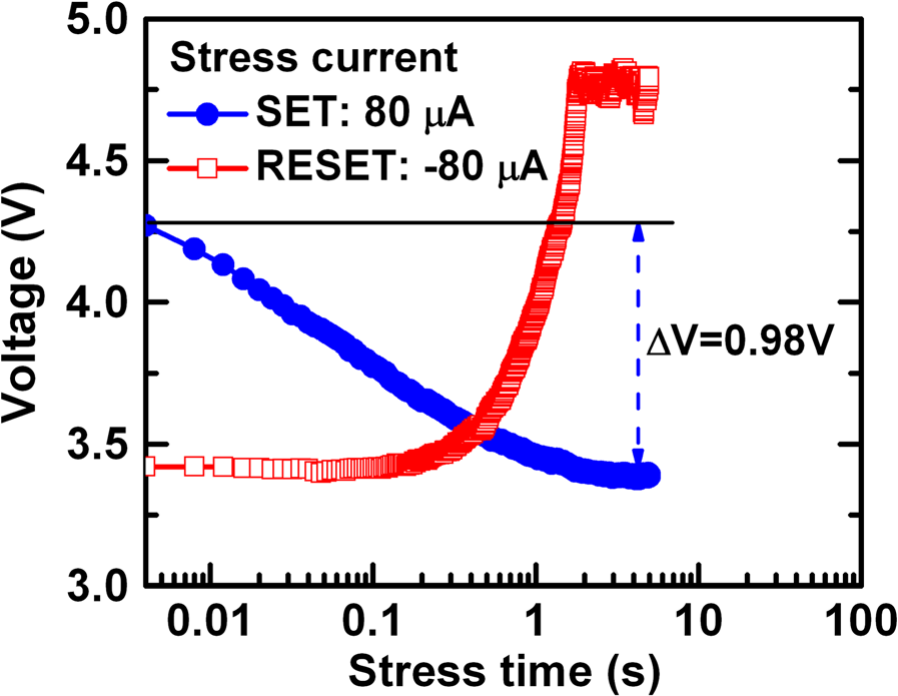
**Voltage shift vs. stressing time under a current of ±80 μA for SET/RESET operations.** The conducting filament diameter is calculated to be approximately 3 nm.

**Figure 8:**
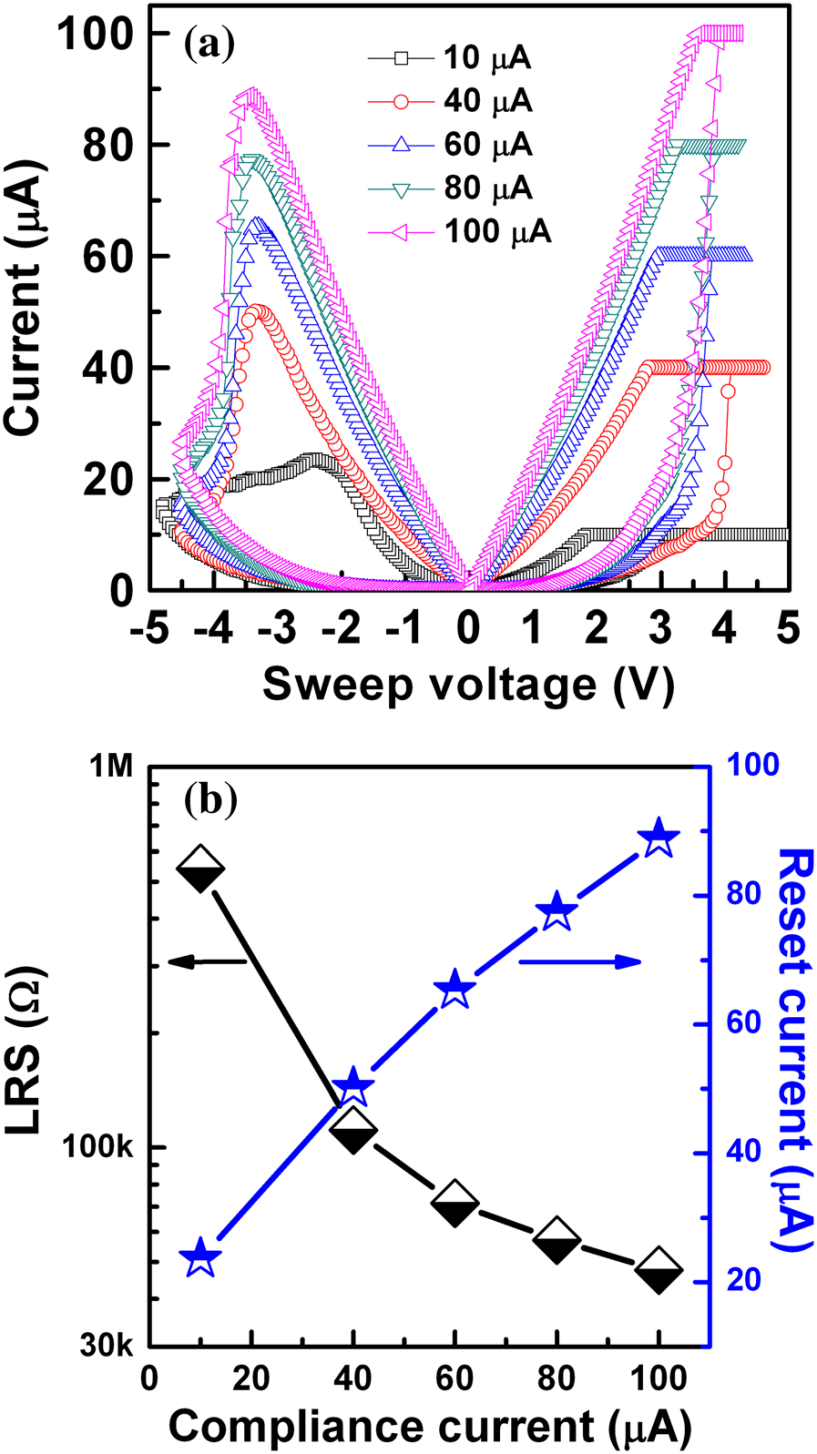
***I*-*V *hysteresis characteristics of (a) LRS and RESET currents (b) with 10- to 100-μA CCs.** A device could be operated with a low RESET current of 23 μA.

**Figure 9:**
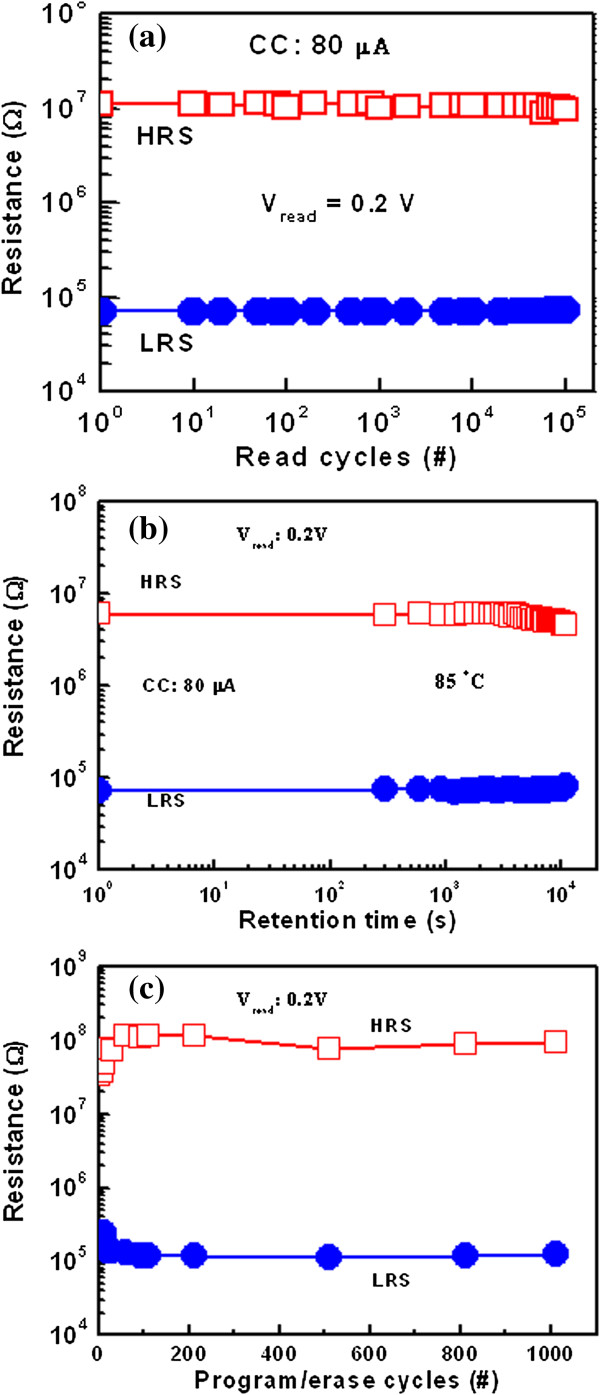
**Long pulse endurance and good data retention. (a)** Long read pulse endurance of >10^5^ cycles and **(b)** good data retention of >10^4^ s with a good resistance ratio of >10^2^ at 85°C are obtained at a low CC of 80 μA. **(c)** Program/erase endurance of >1,000 cycles with a pulse width of 500 μs.

The conducting filament diameter is estimated using a new method under a constant current stress of 80 μA (Figure 7). The voltage decreases (or increases) under positive (or negative) current stress after a SET (or RESET) operation. First, it is considered as a parallel plate metal-insulator-metal (MIM) capacitor. Under external constant current stress, the Ta-O bonds break and create the defects due to oxygen ion migration, which results a reducing voltage across the capacitor. The captured cross section of the defects will lead to the diameter of the conducting filament. Assuming a single cylindrical nanofilament, the diameter (*D*) under SET can be estimated as [47]

(1) $$D=\sqrt{\frac{4qt_{{\mathrm{Ta}}_{2}O_{5}}}{\pi \epsilon_{{\mathrm{Ta}}_{2}O_{5}}\Delta V}},$$

where Δ*V* (changes in the voltage shift under SET and RESET) is found to be 0.98 V (Figure 7), *q* is the electronic charge (1.602 × 10^−19^ C), and $\epsilon_{{\mathrm{Ta}}_{2}O_{5}}$ is the dielectric permittivity of amorphous Ta_2_O_5_ film ($\epsilon_{{\mathrm{Ta}}_{2}O_{5}}$ ≈20 to 25). Considering all values in Equation 1, the diameter of the nanofilament is approximately 2.9 to 2.6 nm. This suggests that the present resistive switching memory device can be scaled down to <3 nm. Previously reported diameters of 5 to 10 nm for Pt/TiO_2_/Pt [12], ≈15 nm for Ti/Fe:SrTiO_3_/Nb:SrTiO_3_[42], and ≈ 1,000 nm for Pt/CuO/Pt [48] are slightly closer and higher than our calculated values, likely owing to the use of different structures as well as materials. Further study may be needed to clearly understand these results. Figure 8a shows the resistive switching characteristics with different CCs from 10 to 100 μA. The low-resistance state decreases with increasing CCs from 10 to 100 μA (Figure 8a,b), which will be useful for multi-level data storage applications. As the filament diameter increases with higher CCs, the low-resistance state decreases, and the value of RESET current increases. The RESET current can be scaled down to 23 μA at a low CC of 10 μA, which will be useful to a low-power operation RRAM in the near future. Our novel device also has a long read pulse endurance of >10^5^ cycles (Figure 9a) and excellent data retention of >10^4^ s with a good resistance ratio of >10^2^ at 85°C at a low CC of 80 μA (Figure 9b). The HRS is slightly decreased with longer elapsed time; however, it is still high, approximately 10 MΩ. Further study is needed to clarify this issue. A data retention of >10^3^ s is also observed for a low CC of 10 μA (not shown here). This RRAM device shows good program erase endurance of >1,000 cycles with a pulse width of 500 μs (Figure 9c). Considering the obtained nanofilament diameter of approximately 3 nm, a high-density (≈100 Tbit/in.^2^) nanoscale nonvolatile memory can be achievable in the future.

## Conclusions

Improvement in resistive switching performance, particularly 10,000 consecutive switching cycles with tight distribution in HRS/LRS of >10^2^, long read pulse endurance of >10^5^, and good data retention of 10^4^ s at 85°C, have been achieved under a low CC of 80 μA by exploiting the oxygen-getter nature of a Ti nanolayer in a W/TiO_*x*_/TaO_*x*_/W structure. A small device of 150 × 150 nm^2^ and a defective TaO_*x*_ film are confirmed by TEM. O^2−^ ion migration because of lower barrier height for electrons leads to a switching mechanism based on filament formation/rupture. The presence of controllable oxygen-deficient TaO_*x*_ nanofilament after switching has been investigated by AES. Furthermore, the device could be operated with a small RESET current of 23 μA. A small nanofilament diameter of 3 nm under a low CC of 80 μA has been calculated using a new method, which has a high memory density of ≈ 100 Tbit/in.^2^, expected to be very useful for future sub-10-nm applications.

## Competing interests

The authors declare that they have no competing interests.

## Authors’ contributions

AP carried out this research work under the instruction of SM. Fabrication process was also instructed by HCC and CSL. AES spectra were taken by TCT under the instruction of SM. All authors read and approved the final manuscript.
